# The Role of Free Tissue Transfer in Merkel Cell Carcinoma of the Head and Neck

**DOI:** 10.1155/2012/742303

**Published:** 2012-08-15

**Authors:** Aldo V. Londino III, Brett A. Miles

**Affiliations:** Department of Otolaryngology, The Mount Sinai Hospital, New York, NY 10029, USA

## Abstract

Merkel cell carcinoma (MCC) is an uncommon neuroendocrine malignancy with a propensity for the head and neck. It typically presents asymptomatically in elderly Caucasians and is characterized by early local and regional spread. There is currently limited data on the appropriate algorithm for treatment of MCC. However, multimodal therapy with wide surgical excision with or without radiation therapy has become standard of care. The location of the primary tumor and intensive adjuvant therapy is often required, provides a challenge to the reconstructive head and neck surgeon. Occasionally, free tissue transfer reconstructive techniques are employed in the reconstruction of MCC defects. This paper will discuss the role of free tissue transfer as a reconstructive option after surgery for advanced head and neck MCC.

## 1. Introduction

Merkel cell carcinoma, originally described by Toker in 1972 [1], is a rare cutaneous malignancy of unknown etiology most commonly seen in the elderly Caucasian population. Incidence has been reported at 0.15 cases per 100,000 in 1986 and 0.44 cases per 100,000 in 2001, with increasing incidence attributed to longer average lifespan and advances in diagnostic technology [2]. MCC is more common in people of advanced age with history of malignancy, immunosuppression, or significant ultraviolet exposure. Recently, infection with the Merkel cell polyomavirus has been shown to be a significant risk factor as well [3–5]. Patients typically present with a firm, painless purplish nodule on the face or upper extremities. Metastatic disease at the time of presentation is rare. Diagnosis relies on tissue biopsy and examination with electron microscopy and immunohistochemical staining. Staging is based upon the traditional TNM (Tumor-node metastasis) classification system with prognostic data showing decreased overall survival with increasing stage of disease [6, 7]. Oncologic surgery within the head and neck presents unique problems, especially when treating Merkel cell carcinoma. Because of the rarity of MCC, there is limited clinical data to guide management. Currently, there are limited clinical guidelines for MCC; however most surgeon clinicians endorse multimodal therapy with wide local excision of the primary tumor and definitive treatment of any clinically significant nodal disease, either with lymphadenectomy or radiation therapy [8]. The role of chemotherapy is unclear, traditionally reserved for diffusely metastatic and/or recurrent disease. Novel targeted therapies are currently being developed and will be discussed elsewhere in this special issue on MCC. Wide excision is currently the standard of care for addressing the primary tumor. However, this can be both functionally and aesthetically devastating in larger lesions. This paper will describe the management options for advanced Merkel cell carcinoma as well as the principles of reconstruction when free tissue transfer is utilized.

## 2. Initial Management

Surgery is the current standard for locoregional disease and has been shown to confer a survival benefit in MCC [8–14]. However, controversy exists regarding the necessity for wide surgical margins, which can be problematic in the head and neck region [9]. The advent of Mohs microsurgery has made definitive oncological resection more precise. Data regarding Mohs surgery in MCC shows comparable, if not superior, local control when compared to traditional surgical excision [15]. As MCC is predominantly found on the sun exposed areas of the head and neck, any surgical intervention will have aesthetic and functional implications. Mohs micrographic surgery provides a more conservative surgical approach while obtaining negative margins and this conserves local tissues. This allows the reconstructive surgeon more options when assessing the most appropriate reconstructive technique. In addition, it has been reported that traditional surgery for MCC frequently results in unrecognized positive deep margins. With Mohs surgery, complete excision is more likely; and local recurrence after reconstruction decreases [16]. In general, lesions on the sun-exposed areas of the face, head and neck, are typically managed with surgical excision using Mohs surgery with local tissue reconstruction. Larger primary tumors may require traditional surgical management for a variety of reasons. Mohs micrographic surgery, while accurate, is not ideal for large extensive lesions requiring general anesthetic for ablative surgery and reconstruction. Large lesions may render Mohs surgery impractical due to the length of the procedure or depth of invasion. In these situations traditional surgical excision is the preferred technique. It should be noted that the author has used a combination technique with Mohs micrographic confirmation of negative cutaneous margins prior to traditional wide local excision of the primary tumor and reconstruction. This allows for rapid excision of the primary lesion with Mohs micrographic control of the cutaneous margins, which improves accuracy and minimizes unnecessary extension of soft tissue margins. The downside to this technique is the inability to assess the deep margins via the Mohs technique and traditional frozen section must be utilized. Regardless of the surgical technique required for excision of the primary tumor, every effort should be made to obtain negative margins.

 Frequently, adjuvant radiotherapy is employed in the management of advanced MCC. Large extensive lesions at risk for local recurrence should be considered for postoperative radiotherapy regardless of surgical margin status. There is data suggesting radiotherapy in addition to Mohs surgery results in improved locoregional control when compared to Mohs surgery alone [16]. Primary radiotherapy may be employed in patients with inoperable tumors or comorbidities significant enough to preclude surgery. Veness et al. presented data on 43 patients treated solely with radiation therapy to the primary tumor. They report an in-field control rate of 75%. However, the majority of patients (60%) go on to have out-of-field metastasis [17]. The determination of appropriate treatment fields for postoperative radiotherapy remains controversial and should be determined by the radiation oncologist after evaluating the patient and operative results. Additional information regarding radiotherapy for MCC will be presented elsewhere in this special issue.

 At present, the recommendation for management of lymph node disease in MCC depends on clinical presentation. For clinically significant lymph node extension cervical lymphadenectomy or therapeutic radiation therapy is indicated after histological confirmation [13, 14]. The role of intervention in clinically negative regional nodal disease is controversial. There is data suggesting that the size of the primary tumor correlates with the risk of occult disease and that occult disease is unlikely with a primary tumor less than or equal to 1 cm [18]. Sentinel lymph node (SLN) biopsy has become a useful tool in attaining a reliable histological indicator of nodal spread and limited data shows decreased recurrence rates where regional management was influenced by sentinel lymph node biopsy. At present, there is insufficient data to determine standardized guidelines for SLN or elective lymph node dissection in the clinically negative neck for MCC. 

## 3. Reconstructive Management

For the majority of surgical defects in the region of the head and neck, locoregional reconstruction with skin grafting or local flaps is functionally and aesthetically adequate. A comprehensive review of factors, which influence the reconstructive approach, is beyond the scope of this paper and will be reviewed elsewhere in the special issue. The extent of disease, viability and quality of surrounding tissue, involvement of the adjacent structures, and history of prior surgery or radiation therapy can make locoregional reconstruction less appealing or impossible. Given the importance of negative surgical margins in MCC, the oncologic surgeon must consider the implications of the risk of local recurrence and consider the propensity for multiple synchronous tumors as well as immune system dysfunction in elderly patients with extensive disease. In these situations, conservative surgery may not be possible, resulting in significant ablative defects of the head and neck. In these situations, free tissue transfer can provide large volume, healthy tissue for reconstruction of the surgical defect, with favorable aesthetic and functional outcomes.

 The increased application of microsurgical reconstruction has resulted in several options for free tissue transfer for soft tissue defects of the head and neck. The anterolateral thigh (ALT) flap, latissimus dorsi, rectus abdominis, scapula/parascapular, and radial forearm flap (RFFF), have been employed for soft tissue reconstruction of extensive defects [19]. A variety of osteomyogenous, or osteocutaneous options exist if bone reconstruction is required. Flap selection depends on the tissue components of the defect (i.e., skin, muscle, and bone) as well as the location, size, depth, and surrounding tissue color/contour. Other aspects during reconstruction such as facial nerve involvement, availability of donor vessels, and donor site considerations such as peripheral vascular disease may alter the reconstructive plan. When considering MCC specifically, the data shows frequent local recurrence as well as a propensity for vertical invasion with positive deep margins being relatively common [20]. These considerations often result in an extensive soft tissue resection involving underlying fat and muscle. In this case, the flaps mentioned above provide a great deal of bulk, require a straightforward harvest with little donor site morbidly, and provide adequate vascular pedicles to limit the need for vein grafting. For defects in the dura, tensor fascia lata grafting is commonly employed with good results. Donor vessels commonly used are superficial temporal system, facial artery, superior thyroid artery, and transverse cervical system [21, 22]. Donor site selection involves a relatively complicated assessment of the ablative defect, the composition of the defect, and the overall condition of the patient. Options for soft tissue reconstruction will be discussed briefly; however it should be noted that a variety of other techniques (i.e., osteomyocutaneous flaps) may be employed if dictated by the defect. The ability to provide large volumes of well-vascularized composite tissue is the most significant advantage of free tissue transfer techniques, when managing large volume defects related to MCC. 

### 3.1. Anterior Lateral Thigh Flap

**Figure 1:**
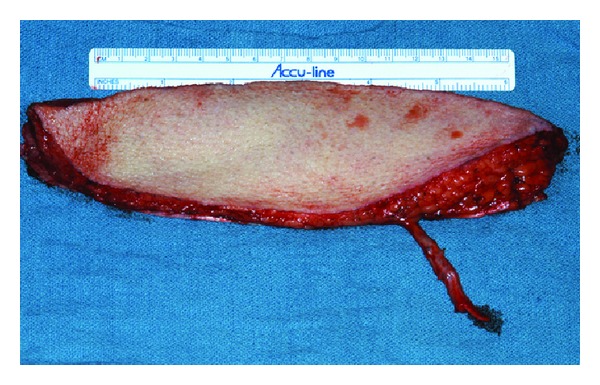
The anterior lateral thigh flap offers excellent soft tissue volume for reconstruction with minimal donor site morbidity. It can be harvested in multiple composite configurations with skin, fascia, and muscle as well as tensor fascia lata. Note excellent pedicle length.

The ALT flap has become a popular reconstructive option for surgical defects within the head and neck. It is easily harvested via a two-team approach, with low donor site morbidity and provides a large softtissue volume, a long and reliable vascular pedicle. The ALT offers the option for dynamic facial nerve reconstruction via motor nerve to the vastus lateralis. The pedicle length limits the necessity for venous grafting and allows microsurgical anastomosis of vessels to occur some distance from the defect [19, 22]. The ALT flap has become the author's preferred workhorse flap for defects >100 sq cm, or in cases where thicker tissue is desired (Figure 1).

### 3.2. Latissimus Dorsi Flap

The latissimus dorsi flap has found use predominantly in reconstruction of scalp defects, especially those with exposed calvarium. It is harvested easily, provides a large surface area with excellent muscle volume and thickness. Donor site morbidity is well tolerated in most patients [21]. Atrophy of the graft provides results in close contour matching with surrounding skin and soft tissue. In most scenarios, this flap should be deepithelialized and covered with a split thickness skin graft for better aesthetic color, matching, and thickness. O'Connell et al. retrospectively evaluated 65 patients with scalp or lateral temporal bone defects, performing a total of 68 free tissue transfers. Based on their experience, the latissimus muscle-only flap with split-thickness skin graft (STSG) provides a large surface area, adequate bulk, and an aesthetically acceptable result in a large majority of scalp defects [22]. Donor site morbidity is generally well tolerated; however in elderly patients who used stabilizing devices such as canes, walkers, or are wheelchair dependent, the ALT may be a more favorable option.

### 3.3. Radial Forearm Flap

**Figure 2:**
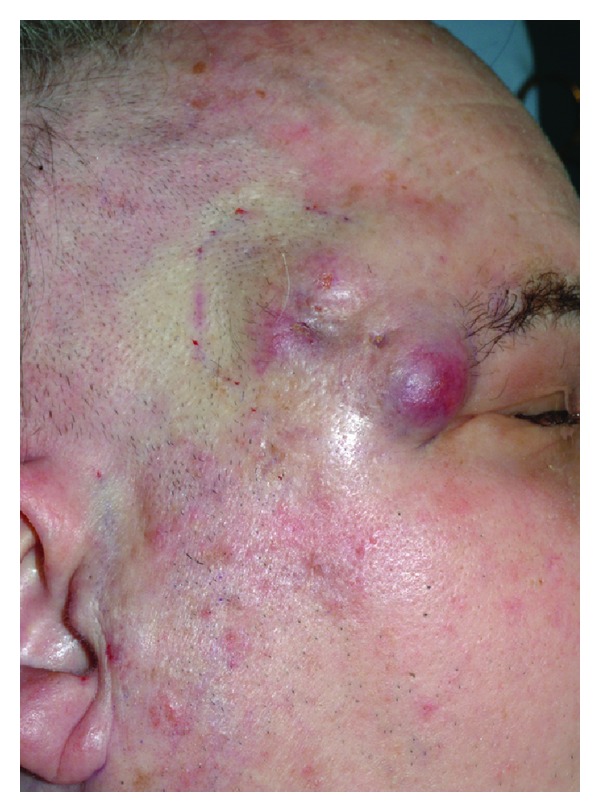
Right-sided Merkel cell carcinoma of the temporal region in a 63-year-old male. The lesion started as a small violaceous nodule approximately six months prior to presentation. This was excised locally at an outside institution and subsequently recurred in the region of the previous excision. The lesion subsequently enlarged and resulted in right-sided weakness in the distribution of the frontal branch of the facial nerve. Pain in the region was mild. Subsequent biopsy revealed MCC. Note multinodular cutaneous induration and anterior violaceous appearance, classic for advanced MCC.

**Figure 3:**
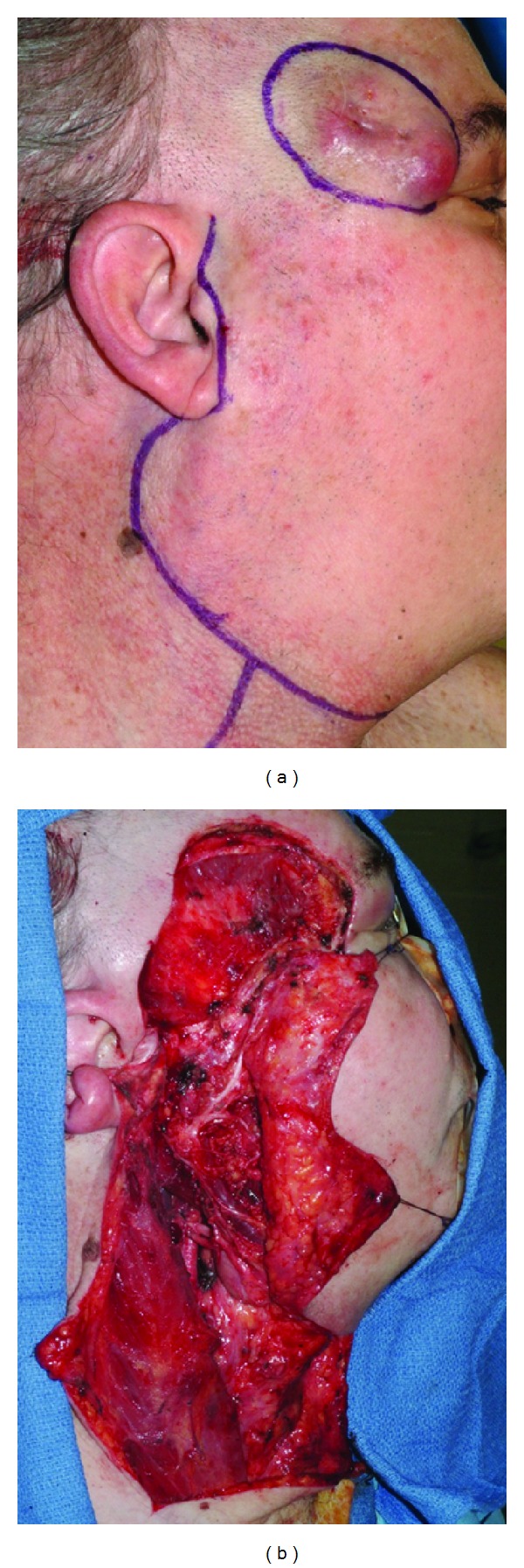
(a) Incision design to allow for wide local excision (1 cm margins) of recurrent MCC with concomitant superficial parotidectomy, selected neck dissection, and microvascular reconstruction. (b) Completion of ablative surgery and cervical lymphadenectomy, noting distal temporal facial nerve involvement. Final pathology indicated negative margin resection. There were no cervical or parotid lymph node metastasis present on final pathologic examination.

**Figure 4:**
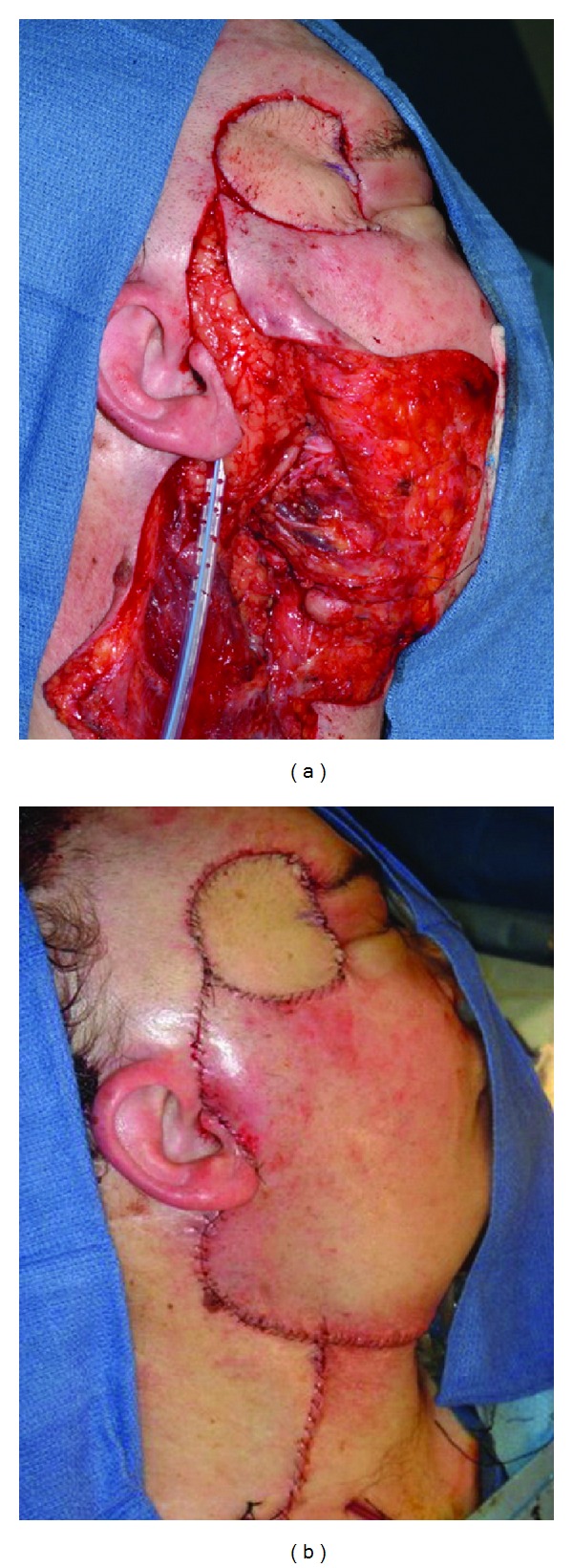
(a) Radial forearm free tissue transfer reconstruction after microvascular anastomosis. (b) Closure. Note excellent skin color match and thin pliable soft tissue in the temporal region avoiding bulky aesthetics and unnecessary lateral canthal traction. Due to the advanced nature of the primary lesion, postoperative radiotherapy was administered to the primary site and parotid bed. At last followup, the patient was alive without evidence of distant disease or local recurrence.

For smaller defects requiring thin and pliable tissue, the radial forearm free flap is preferred. Primarily a fasciocutaneous flap is ideally suited to smaller defects with complex contours where it is desirable to avoid excessive bulk. The reliable pedicle length and predictability of harvest make the radial forearm free flap a commonly utilized reconstructive technique for cutaneous defects of the head and neck. In cases with vessel-depleted necks where veins are unavailable, a semifree radial forearm harvest has been described dissecting the cephalic vein proximally and performing a single arterial anastomosis, which is unique to this flap [23]. Donor site morbidity is well tolerated, however generally inferior to the anterior thigh flap [24, 25] (Figures 2, 3, and 4). 

### 3.4. Rectus Abdominus Flap

The rectus abdominis flap has been used for head and neck reconstruction for cutaneous malignancies and offers the advantage of well-vascularized muscle and large amounts of soft tissue available for reconstruction. When large amounts of muscles are harvested, donor site morbidity is increased and therefore, it is our opinion that other free tissue donor sites offer several advantages over the rectus abdominis flap in the majority of cases. Muscle sparing perforator style flaps may allow for decreased morbidity and superior control of flap thickness, and the deep inferior epigastria artery-based flaps have been shown to be a valid reliable option for head and neck reconstruction [26].

### 3.5. Scapular/Parascapular Flap

The scapular/parascapular flap also has excellent contour and color matching with the forehead and scalp and can typically be closed primarily after harvest with very little donor site morbidity [19, 27]. Harvest may be performed with turned supine positioning and large soft tissue flaps may be harvested. Pedicle length is generally excellent if dissection is performed to the subscapular system. Some surgeons prefer the color and thickness of the scapular/parascapular flap for head and neck reconstruction [27]. In addition, the availability of osseous harvest makes the subscapular system the most versatile flap for complex head and neck ablative defects.

## 4. Facial Nerve Involvement

Merkel cell carcinoma presents frequently on the face and the propensity for vertical invasion often puts the facial nerve at risk. Therefore, there is potential for facial nerve injury secondary to extension of the primary tumor and/or surgical excision for adequate margins (Figure 4). Facial nerve injury can result in lifelong facial asymmetry with profound physiological and psychological consequences, especially in the context of a surgical defect. A discussion of facial nerve reanimation is beyond the scope of this paper; however reconstructive flap selection in free tissue transfer may allow dynamic (latissimus, ALT) or static (ALT, radial forearm/palmaris) reconstructive procedures to be performed simultaneously [28]. Selection of free tissue donor sites should consider the desired facial nerve reanimation strategy in order to minimize additional donor sites.

## 5. Conclusion 

Reconstruction after wide excision of MCC offers several unique challenges including a propensity for elderly patients with poor tissue quality and decreased immune function, large defects and a high probability of disease recurrence, and the risks inherent to adjuvant radiotherapy. For this reason, most reconstructive surgeons favor free tissue transfer as a modality for providing healthy, uninvolved tissue with an acceptable aesthetic outcome. There is clearly a need for higher quality of data in the area, as significant questions remain for the treatment for Merkel cell cancer, as well as the reconstructive methods utilized after ablative surgery. Recently, data has been published suggesting that wide surgical margins during tumor resection may not impact overall survival in patients receiving adjuvant radiotherapy [29]. This is a crucial question for the reconstructive surgeon as it may impact defect size and the probability of local recurrence. Additional data on radiotherapy and targeted chemotherapy for MCC may play a role in deciding the timing for reconstruction, as well as the selected technique. The decision on surgical approach by the reconstructive surgeon should be based on the individual patient and should take into account the details of the clinical scenario in addition to the location and size of the defect. Current free tissue transfer techniques allow the reconstructive surgeon to manage advanced MCC with acceptable functional and aesthetic results, while minimizing morbidity.
